# Fruit transpiration in kiwifruit: environmental drivers and predictive model

**DOI:** 10.1093/aobpla/pls036

**Published:** 2012-11-06

**Authors:** Giuseppe Montanaro, Bartolomeo Dichio, Cristos Xiloyannis, Alexander Lang

**Affiliations:** 1 Department of Crop Systems, Forestry and Environmental Sciences, University of Basilicata, 85100 Potenza, Italy; 2 Sandy Lang Ltd, 402 Muritai Road, Eastbourne 5013, New Zealand

## Abstract

**Background and aims:**

In most fruit crops, storage quality varies greatly between regions and seasons, causing significant commercial loss. Understanding the sources of this variability will contribute to the knowledge of fruit developmental physiology and may also benefit commercial fruit production via altered managements that reduce it or forecasts that predict it. A causal-chain relationship is proposed to help elucidate the sources of variability in fruit storage quality: the weather →(i)→ fruit transpiration →(ii)→ fruit calcium →(iii)→ fruit storage quality. This paper explores the first link of this hypothesis, →(i)→, for Hayward kiwifruit using field measurements of fruit transpiration rate and concurrent meteorological recordings. The aims are to identify the key environmental variables driving fruit transpiration and develop a predictive fruit transpiration model.

**Methodology:**

Fruit transpiration was determined hourly over several 24-h periods by recording weight loss of detached fruit, on Days 23, 35, 49, 65, 94 and 140 after full bloom. Meteorological records were made every 15 min throughout the season at an adjacent regional weather station. A model of fruit transpiration was developed in which the usual meteorological variables (radiation, temperature, windspeed and relative humidity) were incorporated in a Fick's Law transpiration flux equation.

**Principal results:**

Fruit transpiration rate (i.e. the molar flux density, mmol cm^−2^ h^−1^) varied diurnally and decreased during the season. The dominant fruit variable governing transpiration rate was skin conductance and the dominant environmental variables were relative humidity and temperature. Radiation and windspeed were not significantly influential.

**Conclusions:**

The model provides a good fit to the fruit transpiration rate measurements regardless of the time of day/night or the stage of fruit development. The model allows reasonably accurate and continuous predictions of fruit transpiration rate throughout fruit development based on standard meteorological recordings. It also allows estimates of cumulative fruit transpiration throughout the season.

## Abstract (precis)

Seasonal and regional variations in kiwifruit storage quality imply a weather effect. This is perhaps mediated via fruit transpiration and fruit mineral nutrition. Concurrent measurements of fruit transpiration and weather are modelled to predict cumulative fruit transpiration throughout the season.

## Introduction

### Fruit quality and the weather

In most fruitcrop species, key traits such as texture, colour and storage quality are highly variable between regions and seasons. This implies that unidentified aspects of the weather during fruit development somehow impact the expression of these traits at harvest. Identifying the meteorological sources of this quality variability should contribute usefully to our understanding of the physiology of fruit development and it may also suggest altered orchard managements to reduce this variability and/or to provide advance warning of potential fruit quality problems.

### Fruit storage quality and calcium

Fruit are largely phloem fed and, because calcium (Ca) is xylem mobile but phloem immobile, they are generally low in Ca (Taylor and Locascio 2004). As a result, the fruit of many species suffer Ca deficiency disorders (Kirkby and Pilbeam 1984; White and Broadley 2003; Ho and White 2005). There is evidence that kiwifruit fits with this general pattern (Poovaiah *et al*. 1988; Benge *et al*. 2000; Ferguson *et al*. 2003; Thorp *et al*. 2003), although a number of other factors are also known to be involved (Feng *et al*. 2006). Even so, a high fruit Ca content is usually considered a strong positive factor associated with the development of good storage quality in kiwifruit.

### Fruit transpiration and Ca

The regulation of Ca transport in plants is known to be complex (Saure 2005). Nevertheless, because Ca is transported exclusively in the xylem sap and because xylem sap flow is driven by a water potential gradient that develops between any transpiring surface and the roots, it is likely that fruit Ca accumulation will be affected *inter alia* by factors affecting fruit transpiration. These factors are likely to include developmental changes in fruit skin conductance as well as changes in a fruit's aerial microenvironment.

The idea of a direct causal link between a fruit's transpiration and its Ca nutrition is supported in kiwifruit by the twin observations: (i) that most Ca enters the fruit early on in its 5-month development period (Clark and Smith 1988) and (ii) that the conductance of the fruit skin declines markedly during the growth period (Smith *et al.* 1995). The idea also fits with observations for other fruit species such as apples (Jones and Samuelson 1983; Witney *et al*. 1991) and tomatoes (Paiva *et al*. 1998; Taylor and Locascio 2004) where relationships have been found between fruit Ca accumulation and fruit transpiration as affected by fruit microenvironment.

In apple, a number of authors (e.g. Jones and Samuelson 1983; Proctor and Palmer 1991) have found relationships between spur/bourse leaf areas and fruit Ca levels. However, this effect seems to be an indirect one mediated via enhanced xylem cycling (xylem sap flow is commonly from tree to fruit at night, but from fruit to tree in the daytime). Apple bourse and spur leaf transpiration increases the amplitude of this diurnal excursion and this increases the net import of Ca by the fruit (Lang and Volz 1998). Because kiwifruit lacks a comparably close morphological association between fruit and foliage, a similar leaf effect on fruit Ca nutrition is less likely. Therefore, our focus here remains on the simpler and more direct link between a fruit's own transpiration and its Ca import.

### Hypothesis

Combining these ideas, it is hypothesized that the weather influences kiwifruit storage quality by varying fruit Ca nutrition through weather-induced changes in fruit transpiration. This can be expressed in the causal-chain hypothesis: the weather →(i)→ fruit transpiration →(ii)→ fruit Ca →(iii)→ fruit storage quality, where each of the three links →()→ refers to a distinct set of physiological processes. For many fruit species, including kiwifruit, the last of these putative links (iii) has been examined in numerous studies including those cited above, whereas the first two links, (i) and (ii), have not been studied to nearly the same extent. This paper examines the linking processes in (i)—the relationship between the weather and kiwifruit transpiration. A later paper will examine the processes in (ii). While there is a vast literature on the meteorological drivers of foliar transpiration, there are few studies that set out to identify the drivers of fruit transpiration, and none that do this for kiwifruit.

The first aim was to identify the environmental variables that are the principal drivers of fruit transpiration in kiwifruit. The second aim was to develop a model that could predict fruit transpiration at any stage during fruit development and which (for practicability) was based on conventional weather recordings, and on only such crop information as did not require specialist knowledge, much time or expensive instruments.

## Methods

### Experimental site and fruit transpiration measurements

The experiment was carried out in 2006 in southern Italy at the Pantanello Experimental Station (Metaponto, N40°20′ E16°48′). Fruits were taken from mature, Hayward kiwifruit vines (*Actinidia deliciosa* var. *deliciosa*, C. F. Liang and A. R. Ferguson), trained to a pergola system with 625 plants ha^−1^. The vines were regularly microjet irrigated during the season on an approximately weekly basis during May, June and September, and twice weekly during July and August, the months of highest evaporative demand. Bloom was in the third week of May and full bloom was on 23 May. Natural bee pollination ensured reasonably good and roughly simultaneous fruit-set.

Fruit transpiration was assessed gravimetrically by recording the weight loss of detached fruits. Measurements were made hourly over roughly 24-h periods on Days 23, 35, 49, 65, 94 and 140 after full bloom (AFB). The increasing intervals between measurements reflect an anticipated increasing stability in the measured property as the fruit developed.

On each measurement occasion, ∼15 fruiting shoots (each bearing about four fruits) were excised by single cuts at the base. The shoots were immediately defoliated and sealed in plastic bags to reduce water loss, and promptly transferred to the laboratory. Here, at least six fruits were selected for uniformity (size, shape and blemish free) and each was detached from its shoot by cutting its fruitstalk under water (in kiwifruit the fruitstalk comprises both peduncle and pedicel). The cut end was immediately dipped into a 1.5-mL plastic vial containing distilled water. Except for the cut surface, fruitstalks were smeared with a thin film of Vaseline (petroleum jelly) to prevent capillary tracking of distilled water and the vial was sealed with Parafilm to minimize direct evaporative loss of the distilled water. Although a preliminary experiment (not presented) showed that 24-h-old fruit had similar water-loss rates to freshly harvested fruits, a new set of six fruits was sampled and prepared every 3h as described above. This additional work was carried out to avoid the criticism that the measured water-loss rates might have gradually fallen below those of recently detached fruits, which are presumed likely to most closely reflect those of still-attached fruits.

The fruit weight-loss measurements were carried out at the Metapontum Agrobios-ALSIA meteorological site (a regional weather station located well away from trees and structures likely to modify the measured meteorological variables) at the Pantanello Research Station and conveniently close to both the kiwifruit orchard and the laboratory. Fruits were placed at head height (1.7 m) on a frame constructed immediately adjacent to the weather station. For a number of periods of ∼24 h each, individual replicate fruits were weighed hourly using a 3-point (1 mg) balance (Sartorius, Expert Series ED 323S, Göttingen, Germany) situated in a simple shelter constructed *in loco*. The shelter incorporated wind shielding and there was a massive (∼50 kg) marble block beneath the balance for mechanical stability. Hourly weight loss by the fruit (i.e. transpired water) was calculated per unit of fruit surface area, allowing fruit transpiration rate (*E*) to be expressed as a molar flux density (mmol cm^−2^ h^−1^). Fruit surface area (*A*) was estimated as *A* = *a* + *b* × *π* × fruit length × maximum fruit width, where the two coefficients (*a* = 0.798, *b* = 1.0078) were evaluated earlier (Montanaro *et al.* 2006) by regression (*R*^2^ = 0.97) of measurements of these two major fruit dimensions vs. the areas of peeled skin (planimeter) from 70 Hayward kiwifruit.

### Meteorological measurements

The regional weather station recorded the meteorological variables screen air temperature (*T*) (°C) and relative humidity (*RH*) (%) (50Y sensor, Vaisala, Helsinki, Finland), and also the windspeed (*W*) (m s^−1^) (cup anemometer, W/S sensor model 014A, Met One Instruments, Grants Pass, OR, USA) and the total solar radiation (*R*) (kJ m^−2^) (Li-Cor LI200S Pyranometer, Li-Cor, St. Lincoln, NE, USA). The various sensors were connected to a logger (CR10, Campbell Scientific, Logan, UT, USA) programmed to record all these variables at 15-min intervals throughout the season. From this large body of raw meteorological data, sets of hourly mean values of these variables were calculated to align with the time intervals during the 5-month growing season during which the transpiration of the individual fruit was measured hourly over the selected 24-h periods.

### Fruit transpiration model

The measured variables (*T*, *RH*, *W* and *R*) vary widely and cyclically during any 24-h period, and their variations are highly intercorrelated. In the same way, the rates of many plant processes also vary diurnally. It follows that the rate of any measured plant process (photosynthesis, transpiration, extension growth, etc.) will correlate well with any of the meteorological variables, regardless of whether or not that plant process is actually sensitive to that meteorological variable. This means that mere correlation cannot be taken to imply direct causation. Therefore, to identify which of the meteorological variables are causal in driving fruit transpiration, it is necessary to analyse the dataset using a physical or mechanistic model, rather than an unstructured, empirical one.

Transpiration (*E*) from the surface of a plant organ such as a leaf or fruit is usually described (Nobel 2005) using Fick's Law as
(1)
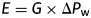

where the value of *E* (mmol cm^−2^ h^−1^) at any instant is proportional to the product of a conductance (*G*) (mmol cm^−2^ h^−1^) and a driving force, the difference (Δ*P*_w_) (dimensionless) between the water vapour (mol fraction) inside the fruit and that in the surrounding atmosphere.

With certain assumptions (see below), the value of Δ*P*_w_ can be calculated from the weather station values of *T* and *RH*. The saturated vapour pressure of water (*p*) (Pa) at *T* is satisfactorily approximated by the expression *p* = 610.7e^17.4^^×^ *^T^*^/(239^^+^ *^T^*^)^ (Goudriaan and van Laar 1994). It is usually assumed (Nobel 2005) that the air spaces inside a plant organ are water saturated so we can write the fruit's internal water vapour pressure (*p*_i_) (Pa) as *p*_i_ = *p*. Meanwhile, the ambient water vapour pressure (*p*_a_) in the surrounding air is given by *p*_a_ = *p* × *RH*/100 (Goudriaan and van Laar 1994). Thus, *p*_i_ – *p*_a_ is the water vapour pressure difference between the inside of the fruit and the surrounding atmosphere. This difference is best written as a mol fraction
(2)
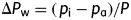

where *P* (Pa) is the atmospheric pressure and Δ*P*_w_ is dimensionless.

There are two possible extensions to the Fick's Law transpiration equation in Equation (1). The first would allow for the influence of a boundary layer conductance (*G*_b_) in series with skin conductance (*G*_s_), where *G* = 1/(1/*G*_s_ + 1/*G*_b_). Boundary-layer conductance is expected to be proportional to *W* (Nobel 2005) and, for common outdoor values of *W*, it is found that for most fruits *G*_b_ ≫ *G*_s_, so that fruit transpiration is dominated by *G*_s_. Note that *G*_s_ is usually lower for a fruit (few or no active stomata) than for a leaf, so *G ≅ G*_s_ and this probably renders the boundary layer extension unnecessary in field situations.

The second possible extension would allow for the elevation of organ temperature (*T*_i_) above that of the ambient air (*T*_a_) under high-radiation (*R*) conditions. In this case, *p*_i_ becomes undefined (unless *T*_i_ has been measured directly) and thus Δ*P*_w_ becomes incalculable. Nevertheless, it is usual (Nobel 2005) for calculations of Δ*P*_w_ to be based on measurements of *T*_a_ alone. Also, in the present case, all our measurements were made over full 24-h cycles, so that values of *R* were either low (early and late in the day) or close to zero (at night time) for much of the time anyway. This renders the assumption *T*_i_ ≅ *T*_a_ more closely true and thus the *T*_i_ ≠ *T*_a_ extension probably unnecessary too.

Notwithstanding these arguments, we will use our dataset to further address the possible influence of *W* on *G* (i.e. *G*≠ *G*_s_) and also the possible influence of *R* on Δ*P*_w_ (i.e. *T*_i_≠ *T*_a_).

### Model identification

One aim in this paper is to find a way to estimate *E* from simple crop data and conventional meteorological data. To do this we should confirm that Equation (1) is indeed the most appropriate model for *E* under the conditions of our experiment (i.e. that the above-mentioned extensions in terms of *W* and *R* are not required). To do this we rearrange Equation (1) as
(3)
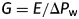



and use this to calculate a set of values of *G* (the set contains 658 values from, roughly, 100 sets of fruit measurements for each of six time periods, i.e. Days 23, 35, 49, 65, 94 and 140 AFB). Next, for each of the six time periods, we regress *G* vs. *W* and, separately, *G* vs. *R* (a total of 12 regressions) and check the slopes to see whether any of these indicates a significant influence on inferred *G*, of either *W* or *R*, respectively.

### Cross-validated estimation of conductance

If we are to use Equation (1) (*E* = *G* × Δ*P*_w_) to estimate fruit transpiration (*E*′) at any time of the season (i.e. at all those times when *E* was not specifically measured), we require not only contemporary records of *T* and *RH* to calculate Δ*P*_w_ (simple weather station data) but also a contemporary estimate of *G*. We know that *G* is strongly dependent on time of season (Smith *et al.* 1995). Here, we estimate *G* at times intermediate between our six periodic measurements by fitting and interpolating the above-mentioned dataset in *G* vs*.* time (days AFB). To measure the accuracy of our interpolated sample estimates of skin conductance (*G*′) we apply a bias-corrected, accelerated bootstrap, cross-validation analysis (Efron and Tibshirani 1993) carried out using SYSTAT 13 (Systat Software, Inc., Chicago, IL, USA). From our full set of 658 vales of *G*, 100 subsamples were selected at random with replacement, each subsample containing 525 values (i.e. 80 % of the total) of *G* derived over the 6 days of measurement.

## Results

### Transpiration

**Fig. 1:**
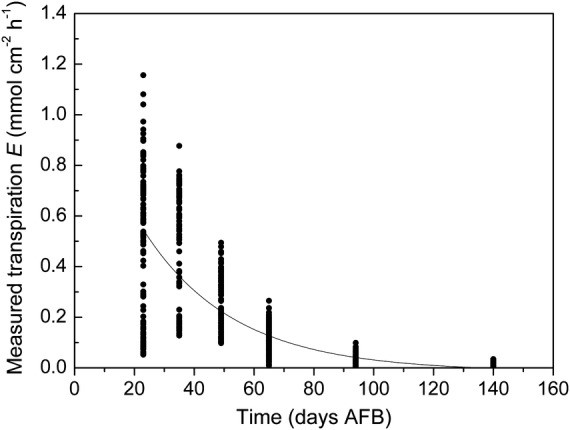
**Fruit transpiration rate (mmol cm^−2^ h^−1^) declines during the season.** The decline reflects a developmental decrease in skin conductance with time after full bloom (AFB). The fitted line is indicative only. The vertical scatter of the hourly transpiration rate values on any particular day is the result of wide diurnal fluctuations in the fruit microenvironment.

**Fig. 2:**
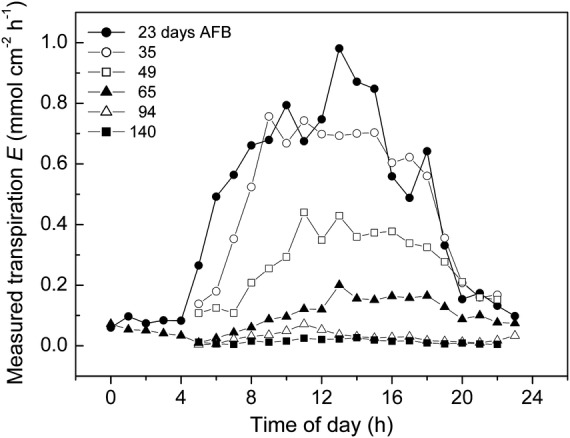
**Fruit transpiration rate (mmol cm^−2^ h^−1^) varies cyclically each day.** The diurnal pattern from near zero predawn to a maximum in the early afternoon is driven by changes in the fruit microenvironment. The seasonal decline is driven by a reduction in skin conductance, e.g. compare Days 23 and 140 AFB in the set of six nested curves.

Fruit transpiration rate (mmol cm^−2^ h^−1^) declines continually during the 5-month developmental period from a spring bloom, through summer, to an autumn harvest (Fig. 1). Diurnal changes in the fruit microenvironment impose considerable variability (the vertical scatter) upon the hourly transpiration rate values measured on any particular day. The nested curves of the same data, but plotted vs. time of day (Fig. 2), show that fruit transpiration rate cycles diurnally, generally taking a maximum value around the middle of each day and reducing to a minimum at night.

### Model identification

Twelve model-identification regressions were carried out (six of *G* vs*. W* and six of *G* vs*. R*). The *P* values of the multiplicative coefficients (the slopes) of these regressions lay between a minimum of 0.098 and a maximum of 0.884 (all were well above the significance threshold of *P* = 0.05), lending no support to the hypothesis that *G* was significantly related to either *W* or to *R*.

### Conductance

**Fig. 3:**
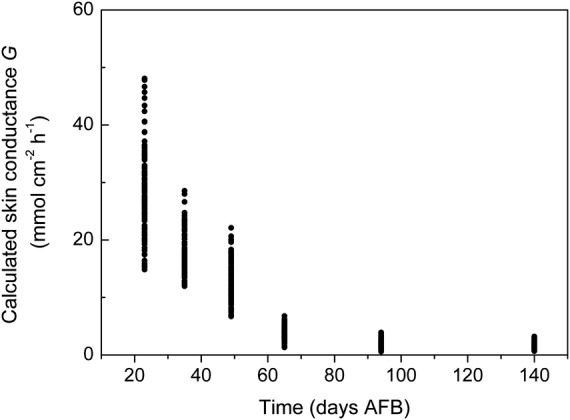
**Skin conductance vs*.* time.** Skin conductance *G* (mmol cm^−2^ h^−1^) is calculated using Equation (3) (*G* = *E*/Δ*P*_w_) from field measurements of *E* (mmol cm^−2^ h^−1^) and from inferred values of Δ*P*_w_ (dimensionless) based on field measurements of *RH* and *T*. Time is in days AFB. Note the sharp, early season decline in *G*.


Fruit skin conductance, calculated from Equation (3), shows a rapid decrease with time in days AFB (see Fig. 3). Estimation of *G*′ at times intermediate between our six periodic measurements involved a cross-validation analysis of the dataset of *G* vs*.* time (*τ*) (days AFB) employing a linear fit to the log : log data (log *G*: log *τ*) to evaluate the exponential model:

(4)
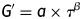



**Fig. 4:**
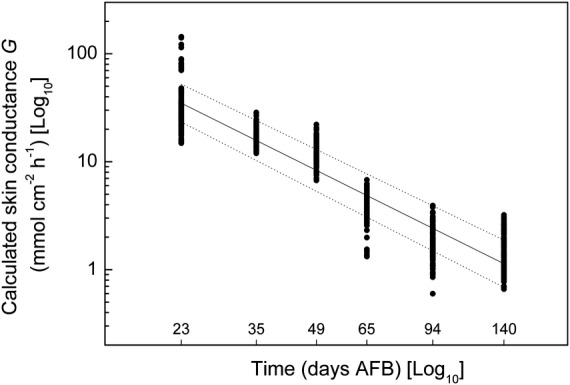
**Log skin conductance vs*.* log time.** The fitted line is the estimated conductance (*G*′) (mmol cm^−2^ h^−1^) vs*.* time (*τ*) in days AFB, using Equation (4) (*G*′ = 13 400 × *τ*^−1.90^) (adjusted *R*^2^ = 0.87). Equation (4) was developed using a bias-corrected, accelerated bootstrap, cross-validation analysis. The upper and lower fitted lines indicate the 95 % CI about the model.

The two coefficient estimates are *α* = 13 400 (95 % CI: lower 10 700, upper 16 800) and *β* = −1.90 (95 % CI: lower = −1.95, upper = −1.84). This model (adjusted *R*^2^ = 0.87) is shown in Fig. 4 as the central straight line in the log : log plot of conductance *G* vs. time in days AFB. This allows us to interpolate for *G*′ at any time between the first (23 days AFB) and last measurement dates (140 days AFB). It should also allow limited extrapolation into the time periods immediately preceding and following these times.

**Fig. 5:**
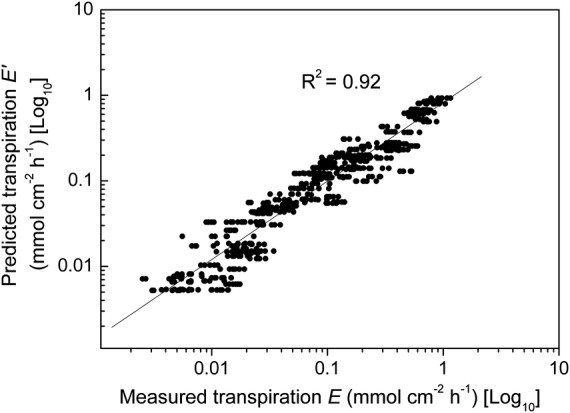
**Predicted (*E***′) **vs*.* measured (*E*) fruit transpiration.** Log *E*′ (mmol cm^−2^ h^−1^) (*y*-axis) values are based on our model and log *E* (mmol cm^−2^ h^−1^) (*x*-axis) values are based on fruit weight-loss measurements. The plotted values to the bottom and left are generally for older fruit (low *G*) and at night (high *RH*, low *T*) and those to the top and right are for younger fruit (high *G*) and in the daytime (low *RH*, high *T*). Linear regression of log *E*′ : log *E* gives a satisfactory fit (*R*^2^ = 0.92) to the exponential *E*′ = 0.8237 × *E*^0.9178^.

To see how well the above model in *G*′ is able to predict fruit transpiration *E*′ from a Hayward kiwifruit, we insert values of *G*′ from Equation (4) into Equation (1) along with the appropriate values of Δ*P*_w_ (calculated from meteorological measurements of *RH* and *T*). The resulting values of *E*′ are plotted (Fig. 5) vs*.* the corresponding set of 658 measured values of *E*. This log : log plot shows a reasonably favourable relationship (*R*^2^ = 0.92) between the predicted and measured *E* values, which has a simple 1 : 1 slope that passes close to the origin. The log : log transformation renders the data more uniformly distributed for regression and also easier to see than the untransformed values, which tend to be crowded towards the origin. Note that for any particular 1-h period, while there is only one value for *E*′ (*y*-axis) there are several values for *E* (*x*-axis) obtained from the ∼6 replicate fruits. Their (horizontal) scatter reflects fruit : fruit variability in *G*.

**Fig. 6:**
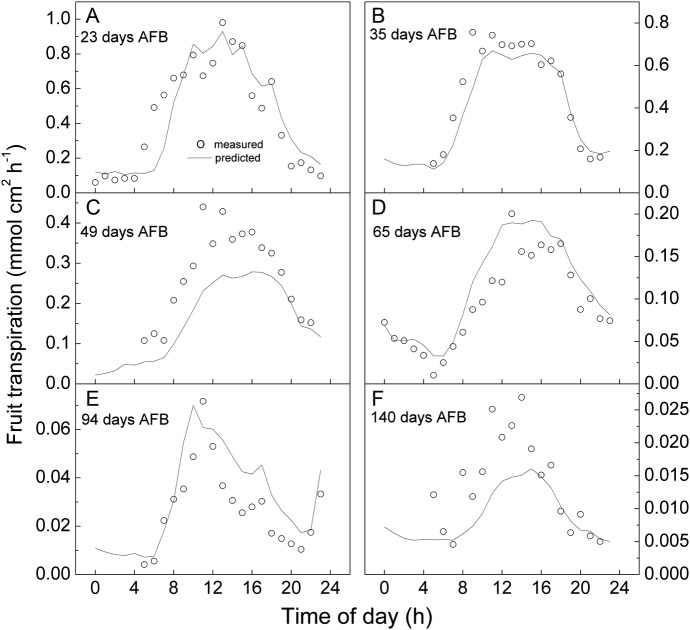
**Comparing predictions with measurements.** Model responsiveness to time of day and to time of season is illustrated by plotting predicted fruit transpiration *E*′ (line) alongside measured fruit transpiration *E* (○) during (A) 23, (B) 35, (C) 49, (D) 65, (E) 94 and (F) 140 days AFB.

To visualize the responsiveness of our model to the time of season (*G* varies considerably with days AFB) and to the meteorological data (*T* and *RH* vary considerably with time of day), it is useful to re-plot the predictions of *E*′ alongside the raw measurements of *E* vs*.* time of day for each of the six measurement periods (Fig. 6).

## Discussion

### Seasonal and diurnal trends

Figures 1 and 2 together indicate that fruit transpiration rates are generally high during early fruit growth but decline to much lower values towards maturity. This decline is not easily attributed to systematic seasonal (spring, summer and autumn) changes in the fruit microenvironment so is more likely due to a developmental reduction in fruit skin conductance (Smith *et al.* 1995). Meanwhile, the regular, diurnal pattern of increases and decreases in transpiration is unlikely to be driven by cyclic changes in fruit skin conductance because kiwifruit have a complex suberized dermal structure with conspicuous hairs but no active stomata (Schmid 1978). Therefore, diurnal patterns of transpiration change are most likely driven by changes in the fruit microenvironment.

### Model identification

On the basis of the arguments that if *W* were significantly to influence *G* (through an effect on *G*_b_) or if *R* were significantly to influence Δ*P*_w_ (through an effect on *T*_i_), then we would expect to see some systematic increase or decrease in calculated *G* with increasing *W* or *R*. From the observation that none of the 12 model-identification regressions had a significant slope we can reasonably conclude that Equation (3) satisfactorily calculates *G*. In other words, that there is no compelling evidence from our data that either *T*_i_≠ *T*_a_ or *G*≠ *G*_s_, so probably that *T*_i_≅ *T*_a_ and *G ≅ G*_s_ and, thus, that Equation (1) is indeed a complete and appropriate model for *E*, and that it requires no extensions in terms of *W* or *R*. This conclusion fits with the expectation that fruit conductance is usually lower than leaf conductance, and so boundary-layer effects are less likely to be significant. It also fits with the observation (Jarvis 1985) that for an isolated leaf or fruit, *R* usually has rather little influence on *E* (the opposite tends to be true for whole-canopy measures).

The magnitude of our field-based evaluations of *G* and the pattern of their rapid early-season decline followed by a period of relative stability are closely comparable to published values of *G* for Hayward kiwifruit (Smith *et al.* 1995) that were measured under laboratory conditions (50 % RH, 20 °C, low light). A regression between these earlier, published values and ours indicates a simple linear relationship that passes close to the origin and has a slope not greatly different from unity (i.e. *G*_Smith *et al*._ = *G*_ours_ × 0.85 + 0.015 (*R*^2^ = 0.97)). This encourages us to believe that our model will not require continual recalibration in terms of *G*.

### Key variables

Our first aim was to identify the key variables influencing fruit transpiration. The results show that throughout fruit development the dominant environmental variables driving transpiration were *RH* and *T* (through Δ*P*_w_), and that the dominant fruit variable was *G*. This simplifying information positions us to develop new physical/physiological explanations for the observed high fruit-to-fruit variability in kiwifruit storage quality in terms of the causal-chain hypothesis proposed here. Moreover, it should be possible to research orchard interventions aimed at regulating to some extent the fruits' ambient *RH* and *T*, and through these *E* (in terms of our fruit transpiration model) and thus fruit Ca and fruit storage quality (in terms of the other links in our causal-chain hypothesis). We envisage interventions such as through the management of orchard shelter, the crop canopy or the irrigation system.

### Transpiration prediction

Our second aim was to develop a quantitative model that could be used to predict fruit transpiration throughout the course of fruit development. The similarity of the predicted (*E*′) and the measured (*E*) values of fruit transpiration rate (Figs 5 and 6) indicates that the model satisfactorily estimates fruit transpiration. That is, the model is well able to accommodate both the large, diurnal fluctuations in fruit transpiration and also its systematic, seasonal decline. It was also considered important for practicability that the model should be based on easily accessible information. It is noted that *RH* and *T* are standard weather station measures with historical data for these variables also being widely available around the world. Meanwhile, the full-bloom date is recorded by most kiwifruit growers.

### Predicting cumulative fruit transpiration

Given the availability of the appropriate meteorological records and the date of full bloom, the model has been able to predict *E*′ at any given instant throughout the season. Nowadays, many weather stations log the main meteorological variables at frequent intervals. This means that taking as its input a semi-continuous stream of meteorological data, the model outputs can be summed and so reported as cumulative fruit transpiration over any desired period. This feature is potentially useful as cumulative fruit transpiration might be expected to track the cumulative import of Ca by the fruit. Moreover, such accumulations should allow comparisons to be made between one region and another or between one season and another (in a manner analogous to the way cumulative ‘growing-degree-day’ information is used routinely in agriculture to account for and to predict biomass accumulations). Such comparisons could help one to identify sources of regional and seasonal variations in fruit quality in terms of our causal-chain hypothesis.

**Fig. 7:**
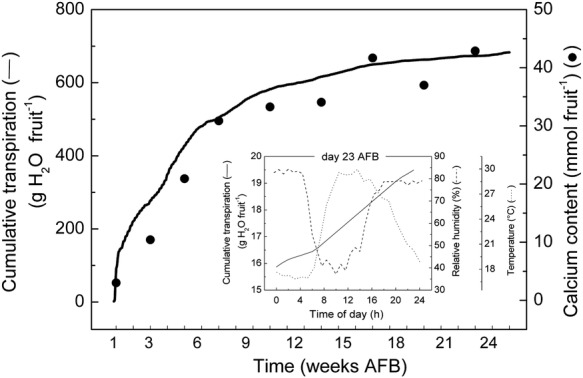
**Cumulative transpiration.** Main graph: Model predictions (solid line) of cumulative fruit transpiration from bloom to harvest. Superimposed (filled circles) are the cumulative fruit Ca content data of Clark and Smith (1988). Note that the shapes of the two cumulative profiles are closely similar. Inset: Hourly predictions of cumulative fruit transpiration (solid line) during Day 23 AFB. Most transpiration occurs between 0800 and 1600 h, and corresponds to a sharp rise in temperature (dotted line) and a corresponding fall in relative humidity (dashed line).

Figure 7 shows the results of applying the model to predict cumulative fruit transpiration over almost the full period of fruit development. Note that we have extrapolated slightly outside the period (Days 23–140 AFB) over which the model was validated. The line (actually a series of ∼15 000 dots plotted at 15-min intervals over ∼5 months) emphasizes the high proportion of total fruit transpiration that occurs early in the season. This character is attributable predominantly to the high early-season values for *G* (see Fig. 3). Interestingly, as foreshadowed, this profile agrees reasonably well with the period over which a kiwifruit accumulates most of its Ca (the published Ca data of Clark and Smith (1988) are superimposed as a series of large dots). The inset in Fig. 7 shows details of the pattern of predicted cumulative fruit transpiration during a single arbitrarily chosen day (Day 23 AFB). This diurnal pattern is generated by cyclic changes in the variables *RH* and *T* also shown.

## Conclusions and forward look

This study develops a model through which the fruit transpiration rate in Hayward kiwifruit can be predicted with reasonable accuracy at any time of the day over almost the entire fruit growth period. Moreover, given a continuous stream of meteorological data, the model can estimate cumulative kiwifruit transpiration for any particular location or season. The work also identifies the key meteorological variables driving kiwifruit transpiration (*RH* and *T*). Thus the model satisfactorily meets its objective of examining the first link, →(i)→, in the casual-chain hypothesis proposed.

The model suggests that it is at least plausible that orchard managements that lower *RH* or raise *T* will increase fruit transpiration and so may also raise fruit Ca more effectively than increasing the Ca availability in the soil (Taylor and Locascio 2004). However, establishing these further links in the causal-chain hypothesis will require additional field research. Nevertheless, if these further links can be successfully established, this study will help to explain the observed variability of kiwifruit Ca content and kiwifruit storage quality between seasons and regions.

We are encouraged to believe that broadly similar results and conclusions will be obtained in similarly structured studies with other important commercial fruitcrop species (apples, grapes, tomatoes, capsicum, etc.). Such studies are expected to give rise to further physiologically and commercially useful information.

## Sources of funding

This research was partially funded by three programmes of the Italian Ministry of University Research: PRIN2005, PRIN2009 and Rientro Cervelli.

## Contributions by the authors

All authors contributed to a similar extent overall.

## Conflict of interest statement

None declared.
